# The Role of Clitoral Anatomy in Female to Male Sex Reassignment Surgery

**DOI:** 10.1155/2014/437378

**Published:** 2014-04-10

**Authors:** Vojkan Vukadinovic, Borko Stojanovic, Marko Majstorovic, Aleksandar Milosevic

**Affiliations:** ^1^University Children's Hospital, Tirsova 10, 11000 Belgrade, Serbia; ^2^School of Medicine, University of Belgrade, 11000 Belgrade, Serbia

## Abstract

*Introduction*. Controversies on clitoral anatomy and its role in female sexual function still make clitoral reconstructive surgery very challenging. We evaluated the role of clitoral anatomic features in female to male sex reassignment surgery. *Material and Methods*. The study included 97 female transsexuals, aged from 18 to 41 years, who underwent single stage metoidioplasty between March 2008 and January 2013. The operative technique involved vaginectomy, the release of clitoral ligaments and urethral plate, urethroplasty by combining buccal mucosa graft and genital flaps, and scrotoplasty with insertion of testicle prostheses. Postoperative questionnaire was used to evaluate aesthetic, functional, and sexual outcome. *Results*. The mean followup was 30 months. The mean length of the neophallus was 7 cm, compared to mean preoperative length of the hypertrophied clitoris of 3.3 cm. Complications occurred in 27.84% of all patients, related mostly to urethroplasty. Voiding while standing was achieved in all cases. None of the patients had problems in sexual arousal, masturbation, or orgasms. *Conclusion*. Accurate knowledge of the clitoral anatomy, physiology, and neurovascular supply is crucial for a successful outcome of female to male sex reassignment surgery. Our approach appears to ensure overall satisfaction and high quality of sexual life.

## 1. Introduction

Throughout history, there have been controversies concerning the anatomy of the clitoris and its role in female sexual function. The first comprehensive evaluation of clitoral anatomy was reported by De Graaf in the 17th century, followed by the study of Kobelt in the 19th century. Cadaver studies and magnetic resonance imaging have recently managed to clarify the exact position, structure, and innervation of the human clitoris [1, 2]. Clitoris is defined as a highly innervated and vascular erectile structure, consisting of the glans and paired erectile bodies—bulbs, crura, and corpora. The intimate relation of these erectile tissues to distal urethra and vagina is believed to have an important role in sexual response. Nevertheless, there are certain aspects of innervation, histology, and physiology of the clitoris that still remain unclear, making our knowledge of the correlation between clitoral anatomy, sexual function, and genital surgery incomplete [3, 4]. Therefore, clitoral reconstructive surgery still poses a great challenge for the genital surgeon, encompassing many different procedures and techniques. Total amputation of the clitoris used to be the single treatment for patients with clitoris hypertrophy (disorders of sexual development, congenital adrenal hyperplasia), causing female genital mutilation. In order to preserve sensation and achieve better aesthetic appearance, more refined techniques of recession and reduction clitoroplasty have been described [5].

Female to male transgenderism is another indication for clitoral reconstructive surgery, where the creation of a neophallus from a hormonally hypertrophied clitoris plays a crucial role. The principles of this technique, as well as the term “metoidioplasty”, were introduced by Lebovic and Laub [6]. Refinements of the technique were described afterwards by other authors. Hage presented his experience in 32 female to male transsexuals, achieving small phallus, that is, hardly capable of sexual penetration [7]. Perovic and Djordjevic reported their metoidioplasty technique, that is, based on repair of the most severe forms of hypospadias and intersex [8]. The main goals of the procedure are male appearance of the genitalia and voiding while standing, without compromising sexual function. We evaluated the principles of metoidioplasty to determine the importance of clitoral anatomy in female to male gender reassignment surgery.

## 2. Material and Methods

**Figure 1:**
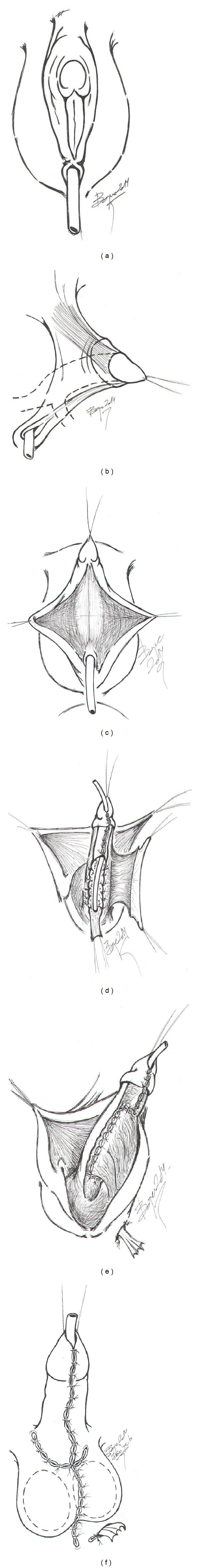
Schematic presentation of clitoral anatomy in metoidioplasty. (a) Ventral aspect of hormonally enlarged clitoris; labia majora appear as scrotums. (b) Clitoral bodies are curved due to dorsal ligamentous support and short urethral plate. (c) Urethral plate is wide and adherent, with visible demarcation to labia minora. (d) Urethroplasty—buccal mucosa graft, previously quilted, combined with well vascularized labia minora flap. (e) Flap is joined with buccal mucosa graft to create neourethra. (f) Final appearance of the neophallus.

**Figure 2:**
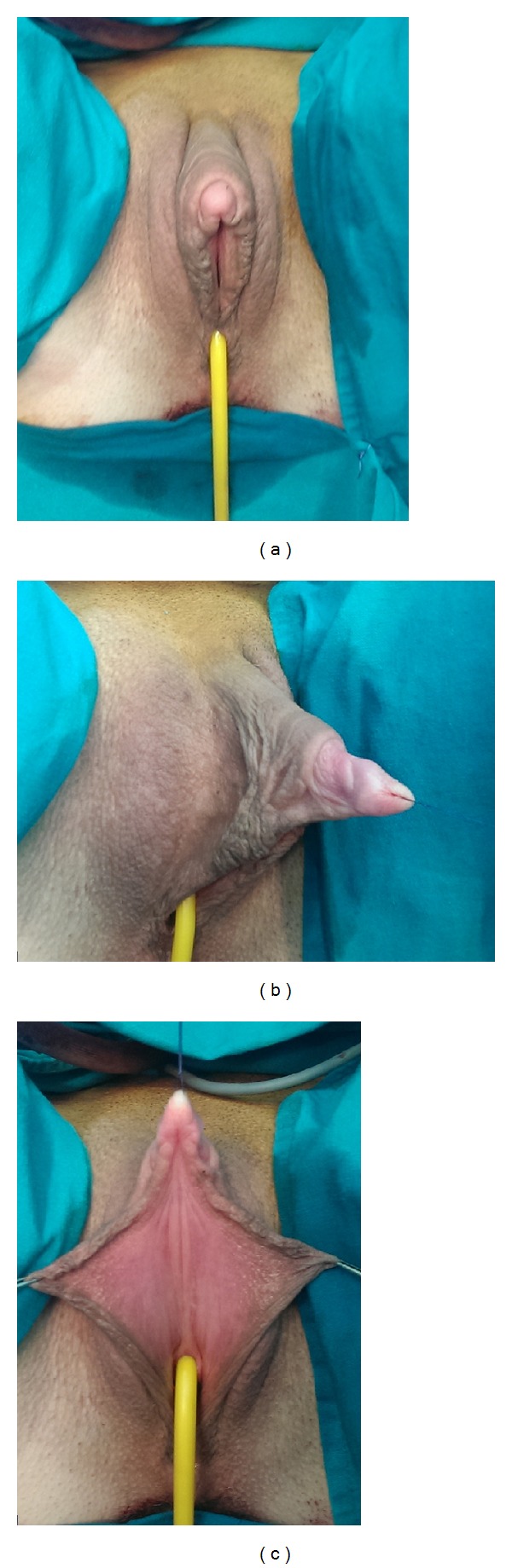
Preoperative appearance of genital area. (a) Ventral aspect. Clitoral bodies and glans are hormonally enlarged. Labia majora resemble scrotums. (b) Lateral aspect of hypertrophied clitoris. (c) Wide urethral plate between the glans and native urethral orifice.

Between March 2008 and January 2013, 97 female transsexuals, aged 18–41 years (mean 29), underwent single stage metoidioplasty, in line with the Standards of Care of the World Professional Association for Transgender Health [9]. After Institutional Ethics Committee approval, informed consent was signed by all patients entering the procedure. The patients were treated hormonally prior to surgery and underwent hysterectomy and adnexectomy either before (63 cases) or simultaneously (34 cases) with metoidioplasty. To achieve additional enlargement of the clitoris, patients were advised to use dihydrotestosterone gel twice a day for a period of 3 months prior to surgery, combined with a vacuum device also twice a day for 30 minutes. Preoperative length of the hypertrophied clitoris ranged from 2.5 cm to 4 cm (mean 3.3 cm). (Figures 1(a), 2(a), and 2(b)).

### 2.1. Operative Technique

**Figure 3:**
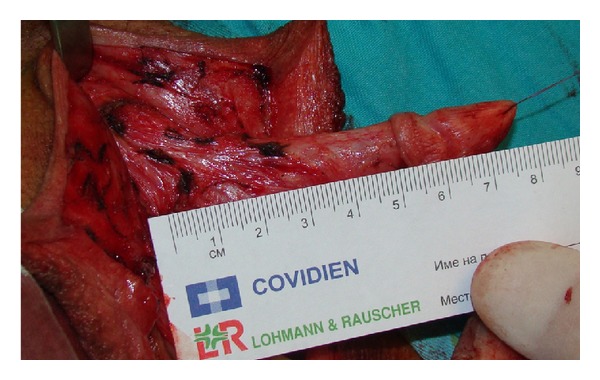
Corpora with glans are 8 cm in length after complete division of clitoral ligaments dorsally and short urethral plate ventrally.

The current operative procedure includes the following steps: vaginectomy, release of clitoral ligaments and urethral plate, straightening and lengthening of the clitoris, urethroplasty by combining buccal mucosa graft and genital flaps, and scrotoplasty with insertion of testicle prostheses. Vaginectomy is performed by total removal of vaginal mucosa (colpocleisis), except the part of anterior vaginal wall near the urethra, which is used for urethral lengthening. Incision is made circumferentially between the inner and outer layers of the clitoral prepuce and continued around the urethral plate and native urethra. After degloving, the clitoral ligaments are completely dissected to bring the clitoris forward. The ligamentous support of the clitoris—suspensory ligament—consists of superficial and deep components. These components are multiplanar, keeping the clitoral body curved. Therefore, its main role is to prevent the clitoris from straightening, while maintaining its stability during sexual activity (Figure 1(b)). Additional curvature is due to short, undeveloped, wide urethral plate, which adheres ventrally to clitoral bodies (Figures 1(c) and 2(c)). In order to straighten and lengthen the clitoris as much as possible, ligamentous components need to be completely released and also urethral plate needs to be divided (Figure 3). Care must be taken to avoid injury of the neurovascular structures during this maneuver. Paired clitoral neurovascular bundle originates from pudendal neurovascular bundle, ascending to the upper part of the clitoral body where the crura unite. The dorsal clitoral nerves pass in large fibers to enter the deep layers of the glans, without visible distal branches that reach the tip of the clitoris. Analogously to glans penis innervation, the innervation of the glans clitoris is impressive, particularly in its dorsal part.

Ventrally, the wide and undeveloped urethral plate is dissected from the clitoral bodies. It is imperative to prevent injury of spongiosal tissue around the urethral plate and excessive bleeding, and also to preserve the blood supply to the urethral plate. Dissection includes the bulbar part of the plate surrounding the native orifice to provide good mobility for urethral reconstruction. Since the wide urethral plate is always short and adheres to the corporal bodies, causing the ventral clitoral curvature, it is divided at the level of glanular corona. This achieves a complete straightening and lengthening of the clitoris.

**Figure 4:**
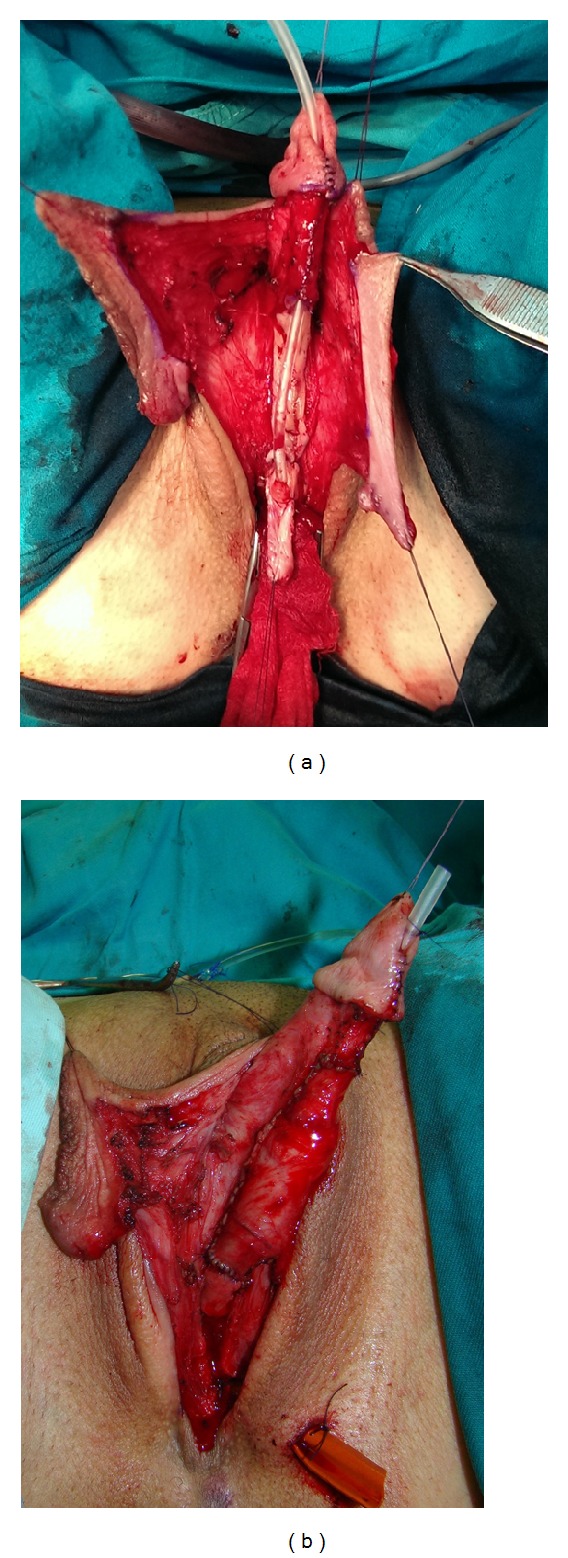
Urethral reconstruction. (a) Flap is harvested from the left labia minora, with preserved vascularization. Previously, buccal mucosa graft is quilted to the ventral side of the corpora. (b) Flap is joined with buccal mucosa graft to create neourethra without tension.

**Figure 5:**
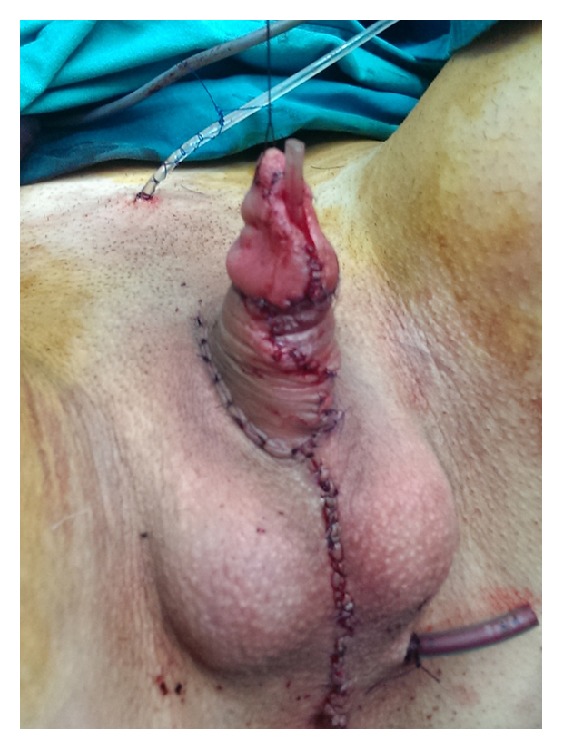
Appearance at the end of surgery. Penile skin reconstructed using remaining genital skin. Two testicular implants inserted into scrotums created from both labia majora.

The bulbar part of urethra is created by joining the flap harvested from anterior vaginal wall and the remaining part of divided urethral plate. Additional urethral reconstruction is performed using a buccal mucosa graft and vascularized genital skin flaps (Figures 1(d) and 1(e)). The buccal mucosa graft is harvested from the inner cheek using a standard technique. The graft is fixed and quilted to the corporeal bodies starting from the advanced urethral meatus to the tip of the glans. Urethra can then be covered using a labia minora flap or dorsal clitoral skin flap. The inner portion of labia minora is dissected to create a flap with appropriate dimensions without dissociation from the outer labial surface. This ensures an excellent vascularization of the flap (Figure 4(a)). The flap is joined with the buccal mucosa graft, without tension, over a 12–14 Fr stent to create the neourethra (Figure 4(b)). Outer surface of the labia minora then covers all suture lines, forming ventral penile skin. In the case of poorly developed labia minora, a well-vascularized longitudinal island flap is harvested from dorsal clitoral skin. The flap is transposed ventrally by the buttonhole maneuver. Lateral edges of the skin flap and the buccal mucosa graft are sutured together with a one-layer running suture to form the neourethra. Abundant flap pedicle is fixed laterally to cover all suture lines of the neourethra. The glans is incised in two parallel lines and both glans wings are dissected extensively, to enable glans closure without tension, creating a conical glans. The penile body is reconstructed using the remaining clitoral and labia minora skin. The labia majora are reconstructed to create the scrotum. Silicone testicle prostheses are inserted through bilateral incisions at the top of labia majora (Figures 1(f) and 5). Suprapubic urine drainage was placed in all cases and kept in for 3 weeks. The urethral stent was removed after 10 days. Postoperative use of vacuum pump is necessary to prevent retraction of the neophallus, starting three weeks after surgery.

A postoperative interview was used for evaluation of aesthetic, functional, and sexual outcome. Structured questionnaire with determined response categories was derived from a structured interview (BVT, Biographical Questionnaire for Transsexuals and Transvestites; Verschoor & Poortinga, 1988), with additional self-created items regarding general sexual life and patients' satisfaction with aesthetic outcome [10]. Self-developed items were measured on a three-point scale ((1) dissatisfied; (2) partially satisfied; (3) completely satisfied). Patients were asked about overall satisfaction with the appearance of their new genitalia and voiding while standing, as well as sexual parameters: quality of erection and erogenous sensation of the neophallus, sexual arousal, frequency of masturbation, orgasm during masturbation, sexual intercourse with partner, and overall sexual satisfaction.

## 3. Results

Followup ranged between 13 months and 69 months (mean 30 months). The length of the neophallus was from 5 cm to 10.5 cm (mean 7 cm). Intraoperatively measured length of the reconstructed urethra was from 9.5 cm to 14 cm (mean 11 cm).

Complications occurred in 27 patients (27.84%). They were classified as minor, which could be managed nonoperatively, and as major, which required additional surgery. Minor complications included dribbling and spraying during voiding and were reported by 17 patients (17.53%). These spontaneously resolved within 3 months after surgery in all cases. The major complications were related to urethral reconstruction and testicular prosthesis. There were 2 urethral strictures (2%) and 6 fistulas (6.18%) that were successfully repaired 6 months later by minor surgical procedures. Normal micturition was obtained in all of these cases, with no urinary leaks. Testicular displacement occurred in 2 patients and was corrected by the replacement and proper positioning of the displaced prosthesis. Reconstruction of the mons pubis region, neophallic skin, or scrotum, due to aesthetic appearance, was performed in 11 patients (11.34%).

Majority of patients (83.50%) were completely satisfied with the new appearance of their genitalia, while 87.63% reported overall complete sexual satisfaction (Table 1). There were no complications related to sexual function. Good quality of erection, sexual arousal, and completely preserved erogenous sensation were reported by all 97 patients, while 70% of patients always experienced orgasm during masturbation. In 20 patients who reported sexual intercourse with partners, length of the neophallus was inadequate for full penetration. Nevertheless, the length of the neophallus was not a limiting factor for voiding while standing, which was achieved in all cases. In 12 patients who additionally required augmentation phalloplasty, microvascular latissimus dorsi muscle flap transfer was performed.

## 4. Discussion

Information on the human clitoris varied over time but was generally overwhelmingly insufficient until recently. As our knowledge of its anatomy and neurophysiology improved through past decades, surgical reconstruction for many indications changed, in order to avoid injury to neurovascular structures, maintain sensitivity of the glans, and achieve good psychosexual and psychosocial outcome [11–13].

Creation of the neophallus is one of the most challenging procedures in female to male sex reassignment surgery. Metoidioplasty has been instituted as a method of choice in female to male transsexuals who prefer avoiding complex, multistaged surgical creation of an adult-size phallus. It is an option in cases where the clitoris seems large enough after hormonal treatment to provide a phallus that will satisfy the patient. Most patients' main desires are to have genitalia of male-like appearance, to be able to void while standing, and to be capable of having a normal sexual relationship [14]. Metoidioplasty is a one-stage procedure with low complication rate, where postoperative appearance and voiding while standing are the key endpoints. The main disadvantage is that it does not produce a phallus long enough to allow penetration, and all patients must be familiarized with this fact prior to surgery. The main goals are straightening and lengthening of the clitoris, as well as reconstruction of the urethra. Clear understanding of the female genital anatomy and sexuality is necessary for a successful outcome [15].

Anatomical background for creation of the neophallus from clitoris was established by reported similarities in the embryology, anatomy, and function of the male and female genitalia. Toesca et al. reported that the corpora cavernosa of the clitoris are essentially similar to the penis, except that there is no subalbugineal layer between the tunica albuginea and the erectile tissue. Consequently, the clitoris can become tumescent but not stiffly erect like the penis. With sexual arousal it becomes engorged, rather than really erect like the penis; however, this fact has no crucial impact on sexual function [16].

Anatomical analogy between fetal clitoris and penis was also observed by Baskin et al. who reported anatomical dissection of the clitoris and its impact on reduction clitoroplasty. The position of the nerves, at 11 o'clock and 1 o'clock along the shaft of the clitoris and glans, was demonstrated. The absence of nerves at the 12-o'clock position and the lowest nerve density on the ventral aspect of the glans were emphasized, as well as abundant innervation of the top and dorsal portion of the glans clitoris [2, 17]. Vaze et al. performed studies on 6 adult cadavers, determining the course of the dorsal nerve of the clitoris [18]. The findings were similar to those of Baskin et al. but the exact function of the dorsal clitoral nerve remained uncertain. It is believed to be a pure sensory nerve, making its role in sexual function unclear. However, it is important to avoid any iatrogenic injury to the clitoral nerves during metoidioplasty. One must take special care when releasing the clitoral ligaments to preserve complete innervation and sensation. Rees et al. facilitated that maneuver by describing, in detail, the anatomy of the ligamentous support of the clitoris in their cadaver dissection study [19]. As their main finding, they described the suspensory ligament of the clitoris, with its superficial and deep components, which was observed in all specimens. The superficial component extends widely from the deep fascia of the mons pubis and attaches the mons pubis to the clitoral body, the full length down the clitoris, entering into the labia majora on their medial aspect. It is a thick, fibro-fatty structure, 7-8 cm wide. The deep component is fibrous and rigid and attaches the clitoral body and bulbs to the pubic symphysis. It appears to be fibrous rather than fibro-fatty, up to 1 cm in thickness. The release of both components of the suspensory ligament, with dissection of the short urethral plate, is the crucial step in straightening and lengthening of the clitoris to create a neophallus in female transsexuals. The length of the neophallus in our patients was from 5 cm to 10.5 cm (mean 7 cm), compared to preoperative length of the hypertrophied clitoris, which ranged from 2.5 cm to 4 cm (mean 3.3 cm).

Reconstruction of the urethra that will enable voiding in a standing position remains one of the main goals of metoidioplasty. In search for better solutions, Djordjevic and Bizic have already reported simultaneous use of buccal mucosa graft and labia minora flap, as a one-stage procedure, with a successful outcome [20]. In this study, we report minor complications in 17.53% of all patients, in the form of dribbling and spraying during voiding. The major complications occurred as urethral stricture and fistula, in 2% and 6.18% of cases, respectively. Voiding while standing was reported in all cases. Also, all patients were satisfied with the new male appearance of their genitalia. In some cases, penile webbing and tissue around the base of the penis presented a problem for voiding while standing, requiring surgical correction.

Maintenance of the sexual function is an imperative for successful outcome of gender reassignment surgery [21]. Certain aspects of clitoral anatomy and neurophysiology, as well as its exact role in female sexuality, are still being assessed. O'Connell et al. evaluated the relationship between the clitoris, urethra, and vagina. Cadaveric dissection and MRI studies brought about an increased understanding of the gross anatomy of the urethra and surrounding erectile tissue. Integral relationship between the clitoris and the distal urethra and vagina is believed to be responsible for female orgasm, making this cluster of tissues (clitoris, distal urethra, and distal vagina) a special entity with a major role in female sexual response [3, 22]. Still, the study of Oakley et al. determined the highest concentration of small nerves within the mucosal surface of the glans, compared to a smaller number of nerve fibers in the skin over clitoral-urethral complex, emphasizing the role of glans clitoris in sexual function [23]. Overall, clitoral glans and clitoral-urethral complex with distal vagina are highlighted as fundaments of female sexual function.

There are recent studies on psychosocial and psychosexual outcome of sex reassignment procedures, but the available literature lacks evidence-based studies, including long-term followup. It is widely accepted that there is greater sexual satisfaction after the transition. Good genital sensitivity is explained by advances and refinements in surgical techniques [14, 21]. De Cuypere et al. evaluated sexual and physical health after sex reassignment surgery, with the mean followup of 6.2 years in female to male transsexuals [24]. They observed improvement of many parameters of sexual life after female to male transition. Sexual satisfaction with a partner after surgery was reported by 81.9% of patients, compared to 50% prior to surgery. Orgasm frequency increased from 45.5% to 77.8%, frequent sexual arousal increased from 40% to 60.9%, and frequent masturbation increased from 20% to 78.3% of participants. Overall sexual satisfaction was reported in 76.2% of the cases, with 19% of unsatisfied patients. Some of these changes can be contributed to the influence of male hormones on sexual behavior and libido, as reported. In our series, overall sexual satisfaction is documented in 87.6% of the cases, orgasm when masturbating is documented in 70%, and erection of the neophallus and sexual arousal is documented in 100%. Hage and van Turnhout reported long-term results of metoidioplasty in 70 patients [25]. Only 8 patients had an uneventful postoperative course, with most complications related to urethroplasty—urethral fistula and stricture. In 17 of 70 patients additional phalloplasty with free flaps was performed at a later stage. Based on their research, metoidioplasty is still the method of choice for female to male transsexuals who are not sure whether they need phalloplasty at the moment of transition.

When performing metoidioplasty, adequate preservation of sexually related tissues is necessary. Urethroplasty and clitoral lengthening and reconstruction, when performed by experienced experts, appear not to compromise sexual function at all. In our study, complete satisfaction with the quality of erection and sensation of the neophallus was reported by all patients. None of the patients had problems or difficulties in sexual arousal, masturbation, or orgasms. Still, long-term analyses on psychosexual and psychosocial outcomes of metoidioplasty are lacking.

## 5. Conclusion

Accurate knowledge of the clitoral anatomy, relations, and neurovascular supply is crucial for achieving a successful outcome in clitoral reconstructive surgery, without compromising sexual function. Metoidioplasty represents a creation of a neophallus from hormonally hypertrophied clitoris in female to male transsexuals. Recent cadaver and MRI studies of clitoral anatomy revealed new features of histological structures and the relationship between the clitoris, urethra, and vagina, as well as the anatomy of its neurovascular supply. These findings, combined with our previous observations, emphasized some aspects of metoidioplasty. Release of all anatomical layers of suspensory ligaments, followed by precise dissection of short urethral plate, is necessary for a complete straightening and lengthening of the clitoris. Preservation of the neurovascular supply, as well as dorsal aspect of the glans, during dissection is essential in maintaining sexual function. An adequate postoperative sexual functioning is reported by the majority of patients. However, analyzing the long-term psychosexual and psychosocial outcome of metoidioplasty is necessary for complete evaluation.

## Figures and Tables

**Table 1:** Patients' satisfaction with metoidioplasty results.

Parameters	Number of patients (%)
Satisfaction with appearance of genitalia	
Completely satisfied	81 (83.50%)
Partially satisfied	12 (12.37%)
Dissatisfied	4 (4.13%)
Voiding while standing	
Completely satisfied	97 (100%)
Partially satisfied	
Dissatisfied	
Quality of erection	
Completely satisfied	91 (93.81%)
Partially satisfied	6 (6.19%)
Dissatisfied	
Erogenous sensation of the neophallus	
Completely satisfied	97 (100%)
Partially satisfied	
Dissatisfied	
Sexual arousal	
(Very) often	97 (100%)
Never—sometimes	
Frequency masturbation	
(Very) often	83 (85.57%)
Never—sometimes	14 (14.43%)
Orgasm during masturbation	
(Almost) always	68 (70.10%)
Never—sometimes	29 (29.90%)
Sexual intercourse with partner	
With penetration	
Without penetration	20 (100%)
Overall sexual satisfaction	
Satisfied	85 (87.63%)
Neutral	7 (7.22%)
Unsatisfied	5 (5.15%)
